# Age-associated B cells are long-lasting effectors that impede latent γHV68 reactivation

**DOI:** 10.1038/s41598-022-25543-1

**Published:** 2022-12-07

**Authors:** Isobel C. Mouat, Iryna Shanina, Marc S. Horwitz

**Affiliations:** 1 Centre for Inflammation Research, University of Edinburgh, Edinburgh, UK; 2 Department of Microbiology and Immunology, The University of British Columbia, Vancouver, BC Canada; 3 Life Sciences Centre, University of British Columbia, Room 3551, 2350 Health Sciences Mall, Vancouver, BC V6T 1Z3 Canada

**Keywords:** B cells, Herpes virus, Virus-host interactions

## Abstract

Age-associated B cells (ABCs; CD19^+^CD11c^+^T-bet^+^) are a unique population that are increased in an array of viral infections, though their role during latent infection is largely unexplored. Here, we use murine gammaherpesvirus 68 (γHV68) to demonstrate that ABCs remain elevated long-term during latent infection and express IFNγ and TNF. Using a recombinant γHV68 that is cleared following acute infection, we show that ABCs persist in the absence of latent virus, though their expression of IFNγ and TNF is decreased. With a fluorescent reporter gene-expressing γHV68 we demonstrate that ABCs are infected with γHV68 at similar rates to other previously activated B cells. We find that mice without ABCs display defects in anti-viral IgG2a/c antibodies and are more susceptible to reactivation of γHV68 following virus challenges that typically do not break latency. Together, these results indicate that ABCs are a persistent effector subset during latent viral infection that impedes γHV68 reactivation.

## Introduction

Humans are infected with an array of herpesviruses that persist within us throughout our lives and require continuous surveillance by the host immune system. Gammaherpesvirus-68 (γHV68) is a murine model of human gamma herpesviruses Epstein-Barr virus and Kaposi sarcoma-associated herpesvirus^1^. γHV68, like other herpesviruses, deploys distinct transcriptional programs during lytic and latent infection, each of which requires a distinct immune response^2^. Age-associated B cells (ABCs) are a unique B cell population implicated in viral infection, autoimmunity, and aging^3–6^. ABCs (CD19^+^CD11c^+^T-bet^+^) are induced following γHV68 infection^7^, though their function throughout the course of infection is not clear. In this study we examine the response of and role for ABCs throughout γHV68 infection, from acute infection through long-term latency.

ABCs were identified in 2011 in the context of female aging and autoimmunity^3,4^ and have since been shown to be increased in an array of viral infections including lymphocytic choriomeningitis virus (LCMV), murine cytomegalovirus, γHV68, vaccinia, human immunodeficiency virus, rhinovirus, SARS-CoV2, and influenza^7–12^. ABCs are elevated in the spleen and circulation during active viral infections and persist primarily in the spleen during chronic infection or upon infection resolution^9,10^. ABCs display multiple functional capacities including the secretion of antibodies, anti-viral cytokines and the activation of T cells^13,14^. T-bet expression in B cells is required for IgG2a/c class switching^15^ and lack of ABCs exacerbates LCMV chronic infection in mice^16^. We predict that ABCs could be playing a role throughout γHV68 infection due to their long-term persistence, activation of T cells, and continuous cytokine and antibody production.

Lytic γHV68 infection is primarily cleared by CD8^+^ T cells^17,18^, following which γHV68 persists in a latent state mainly in previously activated B cells^19^. During latency the immune response persists even though there is minimal production of viral genes and limited production of new virions^20^. Both humoral and cellular responses contribute to controlling γHV68 infection long-term^21,22^. Mice lacking either antibodies or T cells are both able to control γHV68 latency, though mice lacking both antibodies and T cells display elevated γHV68 reactivation^21,23^. IFNγ is critical for controlling γHV68 replication and reactivation from latency^24,25^.

B cells are also known to be important during latent γHV68 infection. B cells act as the primary latent viral reservoir, are required for movement from the lung to the spleen at the onset of latency, and mice deficient in B cells are unable to develop latency following intranasal infection^19,26–28^. B cells secrete anti-viral antibodies long term^29^, but also may inhibit reactivation via secretion of anti-viral cytokines or help to virus-specific T cells. The precise B cell subsets, mechanisms, and factors that facilitate the maintenance of latency and prevent reactivation are not fully understood.

The role of ABCs during acute and latent herpesvirus infection is unknown. Herein, using various tools, we investigate the relationship between γHV68 and ABCs throughout lytic and latent infection. We find that ABCs expand during acute γHV68 infection and persist in the spleen during latency. Well after the initial establishment of latency, ABCs continuously secrete anti-viral cytokines and antibodies and in the face of heterologous challenge, ABCs are required to restrain latent γHV68 reactivation. Thus, our findings highlight a novel role for ABCs as effector cells during γHV68 latency.

## Results

### ABCs are induced and maintained following γHV68 infection in a sex-biased manner

**Figure 1:**
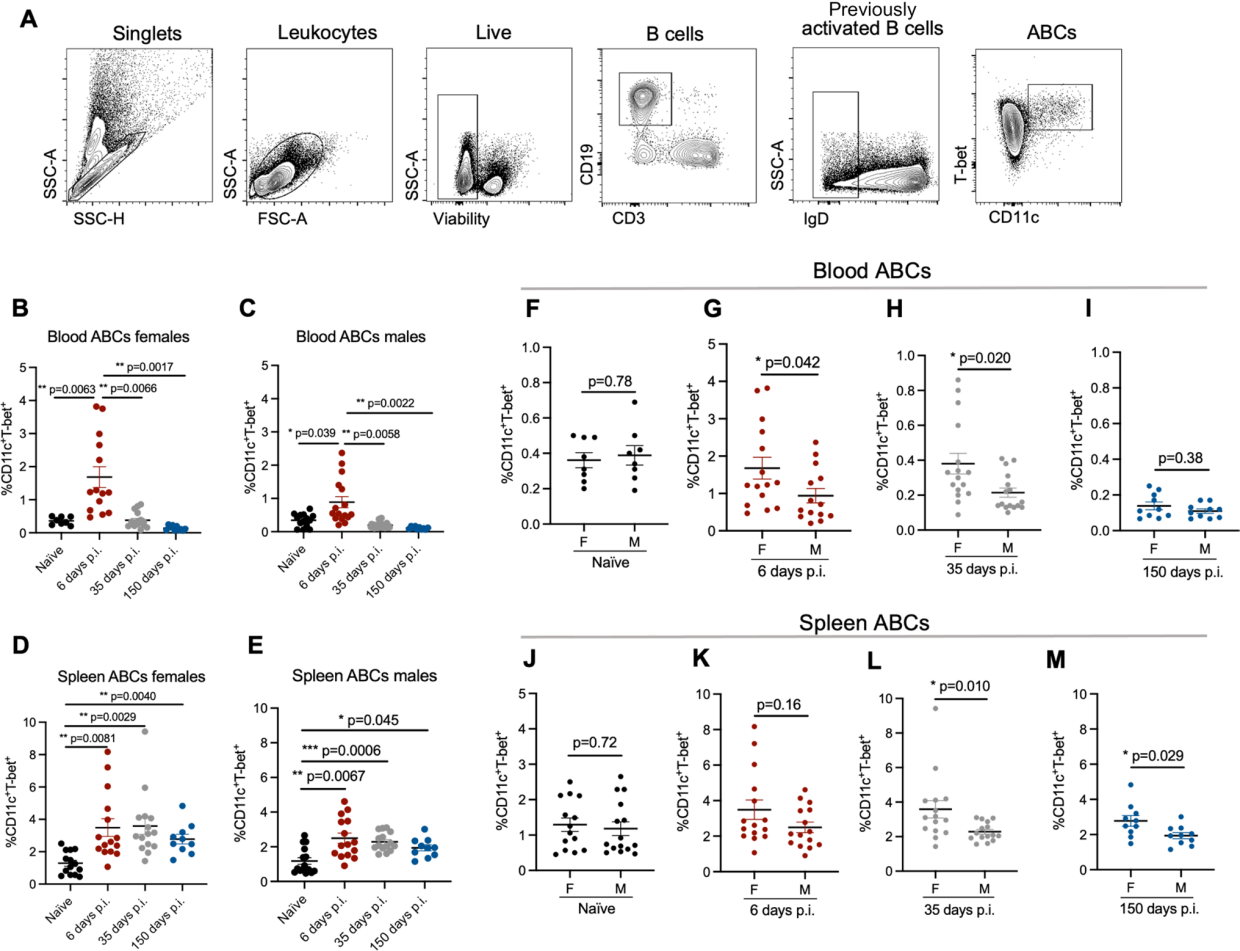
ABCs expand in the blood and spleen throughout γHV68 infection. Blood and spleen collected from C57BL/6(J) mice (6–8-week-old at infection) mock-infected with media (naïve, black circles) or infected i.p. with γHV68 for 6 (red circles), 35 (grey circles), or 150 (blue circles) days and processed for flow cytometry. (**A**) Representative gating strategy of ABCs. IgD, CD11c, and T-bet gating used fluorescence-minus-one (FMO) controls. (**B–E**) Proportion of ABCs (CD11c^+^T-bet^+^) of previously activated B cells (CD19^+^IgD^−^) in the blood and spleen of male and female mice. (**F–I**) Proportion of ABCs (CD11c^+^T-bet^+^) of previously activated B cells (CD19^+^IgD^−^) in the blood or spleen of male and female mice. Same data as presented in panels (**B–E**). n = 10–15 mice per group, data compiled from 4 experiments. Each data point represents an individual mouse. Data presented as mean ± SEM. Analyzed by (**B–E**) one-way ANOVA or (**F–M**) Mann–Whitney test. p-values indicated as asterixis as follows: ***p < 0.001, **p < 0.01, *p < 0.05.

To examine the relative proportion of ABCs during acute and latent γHV68 infection, C57BL/6(J) mice were mock-infected with media (naïve) or infected with γHV68 for 6, 35, and 150 days, blood and spleen were collected and analyzed by flow cytometry to measure ABCs. A gating scheme, described in Fig. 1A, identified CD11c^+^T-bet^+^ as a proportion of previously activated B cells (CD19^+^IgD^−^). We observed the relative proportion of ABCs increased in circulation during acute γHV68 infection (6 days p.i.) compared to naïve mice (Fig. 1B,C). Once latency was well established (day 35 and 150 p.i.), the proportion of circulating ABCs decreased to pre-infection levels (Fig. 1B,C). This indicates that ABCs circulate in response to the lytic γHV68 infection but do not remain elevated in circulation during latency. In the spleen, the relative proportion and total number of ABCs was increased during acute γHV68 infection (day 6 p.i.) relative to naïve mice and remained elevated during latent γHV68 infection at 35 days p.i. and 150 days p.i. (Fig. 1D,E, Fig. S1). These results support previous findings that the spleen is a major site for ABCs following the clearance of acute viral infection^10^.

Intriguingly, we observed that the proportion of ABCs is increased in females as compared to males in both the blood and spleen throughout infection (Fig. 1F–M). Previously, we demonstrated that ABCs display a sex bias during experimental autoimmune encephalomyelitis and at 35 days post-γHV68 infection in the spleen^30^. Here, we have extended this finding and shown that ABCs display female sex bias in both the circulation and spleen during lytic and latent γHV68 infection. While ABCs increase in both females and males following γHV68 infection, there is a sex bias leading to greater expansion in females. These results demonstrate that ABCs are increased in circulation during acute γHV68 infection and continue to endure in the spleen long-term during latent infection.

### ABCs express anti-viral cytokines in response to latent γHV68 infection

**Figure 2:**
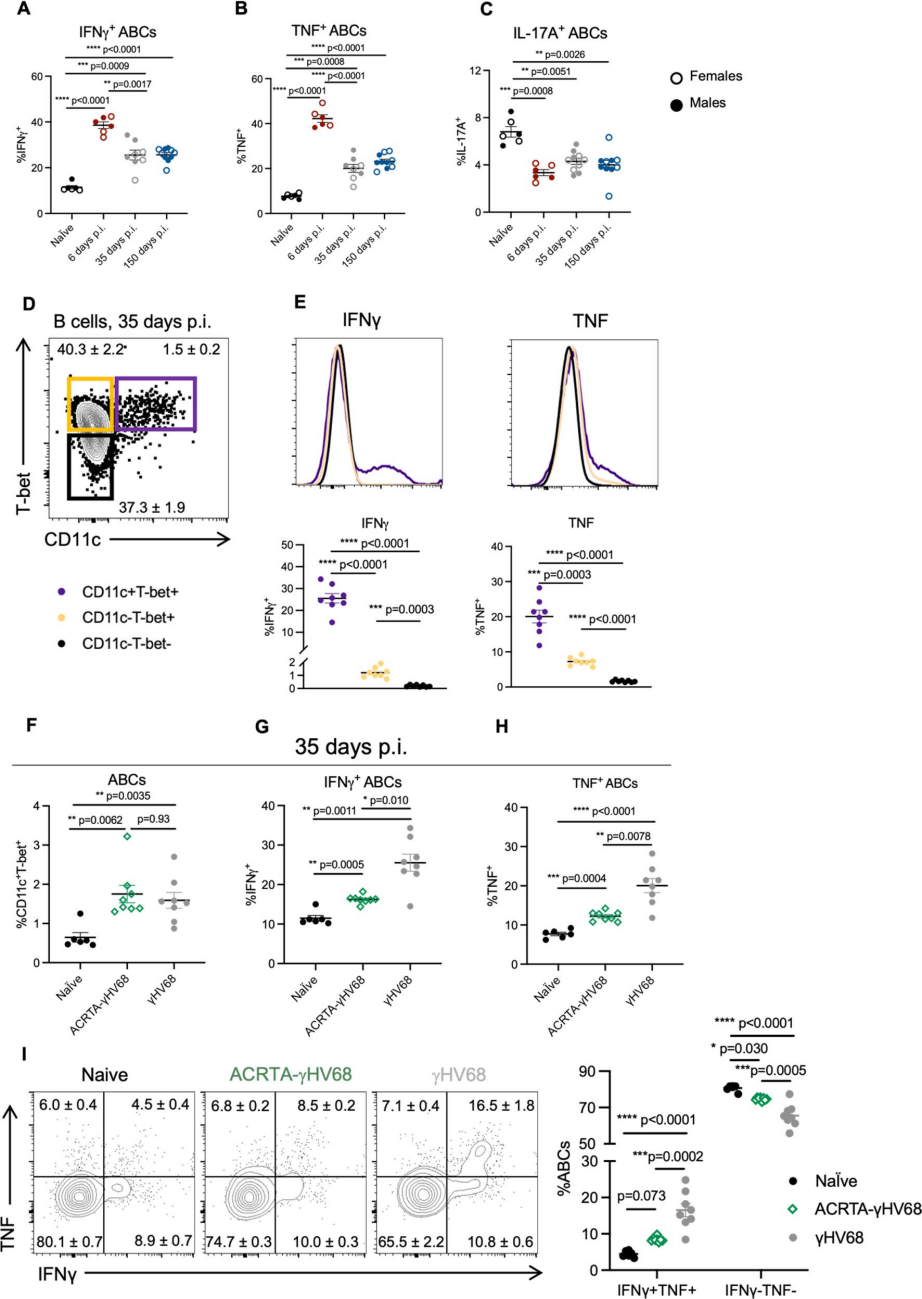
Cytokine production by ABCs in the spleen during γHV68 and ACRTA-γHV68 infection. (**A–C**) C57BL/6(J) mice (6–8-week-old at infection) mock-infected with media (naïve) or infected i.p. with γHV68 for 6, 35, or 150 days. Spleens collected and processed for flow cytometry. Proportion of ABCs in the spleen expressing (**A**) IFNγ, (**B**) TNF, or (**C**) IL-17A, with females presented as open circles and males as filled circles. (**D**) Gating of ABCs (CD11c^+^T-bet^+^) and non-ABC B cells (CD11c^−^T-bet^+^ and CD11c^−^T-bet^−^), previously gated on CD19^+^IgD^−^ cells in the spleen. (**E**) Representative histograms and dot plots of the proportion of ABCs and non-ABC B cells (CD11c^−^T-bet^+^ and CD11c^−^T-bet^−^) expressing IFNγ and TNF from mice infected with γHV68 for 35 days. (**F–H**) C57BL/6(J) mice mock-infected with media (Naïve, black circles) or infected i.p. with γHV68 (grey circles) or ACRTA-γHV68 (green diamonds) for 35 days and spleens processed for flow cytometry. (**F**) Proportion of ABCs (CD11c^+^T-bet^+^) of previously activated B cells (CD19^+^IgD^−^) in the spleen. Proportion of ABCs (CD19^+^CD11c^+^T-bet^+^) in the spleen expressing (**G**) IFNγ or (H) TNF. (**I**) Representative plots and quantification of the proportion of ABCs expressing IFNγ and TNF. Equivalent numbers of females and males per group in all panels. (**A–C**) n = 6–10 mice per group, data combined from two experiments. (**E–I**) n = 6–8 mice per group, representative of two experiments. Each data point represents an individual mouse. Data presented as mean ± SEM. Analyzed by one-way ANOVA. p-values indicated as asterisks as follows: ****p < 0.0001, ***p < 0.001, **p < 0.01, *p < 0.05.

To begin investigating the role of ABCs in the anti-viral response, we measured ABC expression of anti-viral cytokines interferon-γ (IFNγ) and tumor necrosis factor-α (TNF) in the spleen at 6, 35, and 150 days p.i. We found that during acute γHV68 infection (6 days p.i.), roughly 40% of ABCs in the spleen expressed IFNγ and TNF, as compared to about 10% of ABCs in naïve mice (Fig. 2A,B). During latent infection (35 days and 150 days p.i.), a significantly increased proportion of ABCs continued to express IFNγ and TNF compared to naïve mice, though the proportion of ABCs expressing IFNγ and TNF was decreased compared to acute infection (Fig. 2A,B). Throughout the acute and latent infection, we observed a downregulation of IL-17A expression on ABCs (Fig. 2C).

To determine if the level of cytokine expression was distinct from other B cells, we compared the proportion of ABCs expressing IFNγ and TNF to non-ABC B cells. Specifically, we examined expression on ABCs compared to CD11c^−^Tbet^+^ and CD11c^−^Tbet^−^ B cells 35 days post-γHV68 infection (Fig. 2D). A significantly increased proportion of ABCs expressed IFNγ and TNF compared to non-ABC B cells, both CD11c^−^Tbet^+^ and CD11c^−^Tbet^−^ B cells, in the spleen (Fig. 2E). The mean fluorescent intensity (MFI) of IFNγ and TNF, of IFNγ or TNF positive cells, was significantly increased on ABCs compared to both non-ABC populations (Fig. S2A,B). CD11c^−^Tbet^+^ B cells expressed an intermediate level of IFNγ and TNF between ABCs and CD11c^−^Tbet^−^ B cells (Fig. 2E, Fig. S2A,B). Sex differences in ABC cytokine expression were not observed (Fig. 2A–C, Fig. S3A–C). The high level of IFNγ and TNF cytokine expression indicates that ABCs may be functioning in a unique anti-viral capacity during latent infection.

To explore the relationship between latent γHV68 and the ABC population, we infected mice with ACRTA-γHV68, a recombinant strain of γHV68 in which the genes responsible for latency are deleted and a lytic gene, RTA, is constitutively expressed^31^. As a result, γHV68 infection is cleared following acute infection without ever establishing latency. We found that, at 35 days p.i., a time point at which ACRTA-γHV68 is cleared, ABCs are increased to the same level in the spleens of ACRTA-γHV68 infected mice as compared to spleens in mice infected with WT γHV68 (Fig. 2F). Thus, ABCs persist in the absence of latent virus, in a manner similar to memory cells that remain following acute infection.

While the proportion of ABCs remained elevated in the absence of latent virus, cytokine expression was altered. The proportion of ABCs in ACRTA-γHV68-infected mice expressing IFNγ and TNF at 35 days p.i. was significantly reduced compared to those infected with WT γHV68 while remaining significantly elevated compared to naïve mice (Fig. 2G,H). Additionally, the IFNγ^+^ ABCs in mice infected with ACRTA-γHV68 displayed a lower MFI than those from γHV68-infected mice, though the TNF MFI was not significantly different between the two groups (Fig. S2C,D). Without the presence of the latent virus, we observe a decrease specifically in the proportion of ABCs that doubly express both IFNγ and TNF as well as an increase in ABCs that are negative for the expression of both IFNγ and TNF as compared to ABCs from WT γHV68-infected mice (Fig. 2I). This finding indicates that ABCs respond to the latent virus by producing both IFNγ and TNF.

Together, these results show that ABCs are a predominant B cell subset expressing anti-viral cytokines IFNγ and TNF during latent γHV68. Further, our data establish that high levels of expression of these cytokines are dependent on the presence of the latent virus. These findings suggest that ABCs recognize the presence of the quiescent latent virus and respond by expressing anti-viral cytokines.

### ABCs are susceptible to γHV68 infection but are not a major viral reservoir

**Figure 3:**
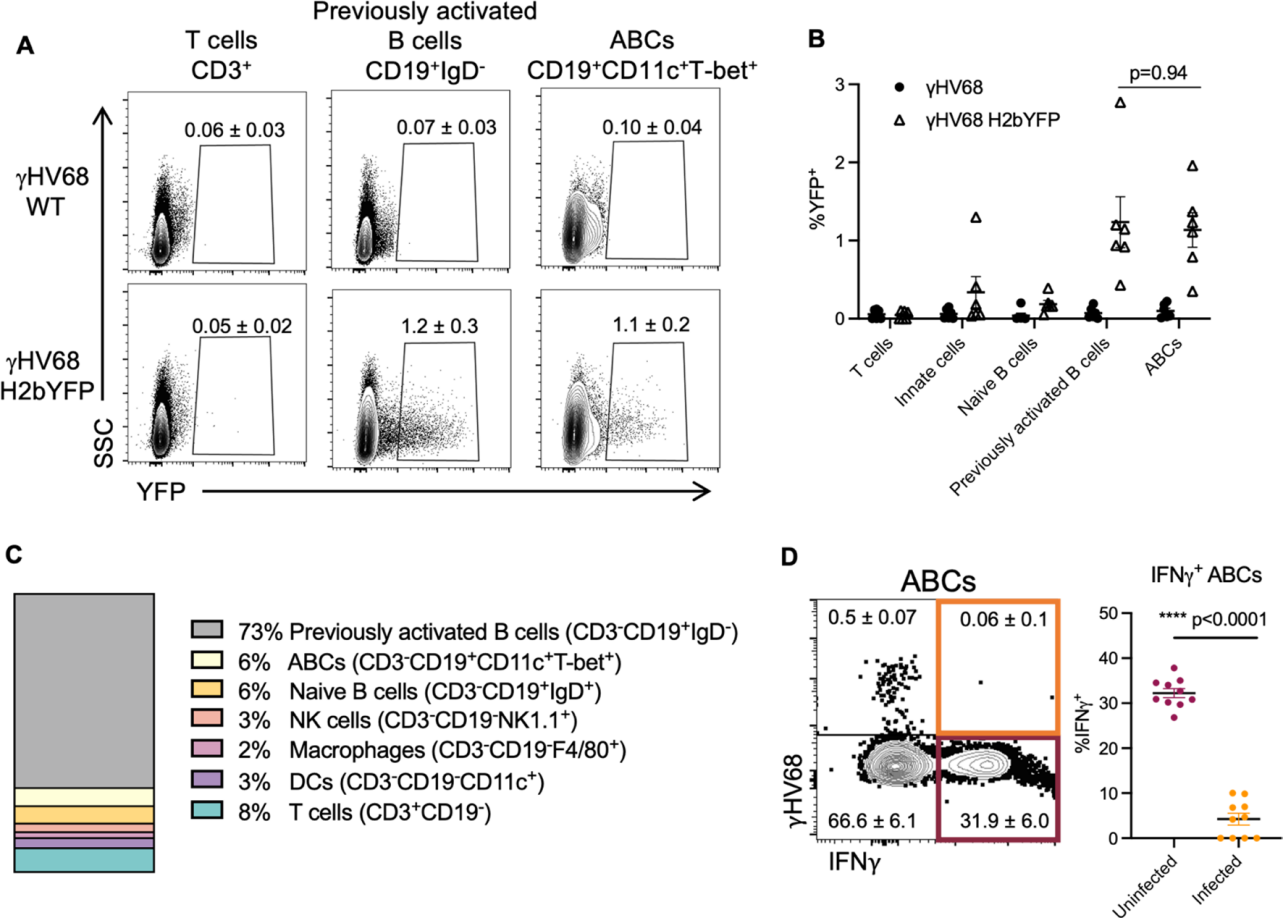
Analysis of ABC infection status with fluorescent γHV68 strain. Female C57BL/6(J) mice (6 to 8-week-old at infection) infected i.p. with γHV68 or γHV68.H2bYFP. 8 days p.i. spleen collected and processed for flow cytometry. (**A**) Representative flow cytometry plots displaying yellow fluorescent protein (YFP) expression (x-axis) versus side-scatter (SSC, y-axis) from mice infected with γHV68 or γHV68.H2bYFP with mean ± SEM. Samples gated on live, single lymphocytes, and then T cells (CD3^+^), total B cells (CD19^+^), previously activated B cells (CD19^+^IgD^−^), or ABCs (CD19^+^CD11c^+^T-bet^+^). Two samples per group concatenated for the purpose of visualization. Representative of two experiments. (**B**) Proportion of cell subsets positive for YFP expression from γHV68-infected mice (filled circles) and γHV68.H2bYFP-infected mice (open triangles). Each data point represents an individual mouse. (**C**) Proportion of YFP^+^ cells that are non-B cells (CD19^−^, white), non-ABC B cells (CD19^+^CD11c^−^T-bet^−^, grey), or ABCs (CD19^+^CD11c^+^T-bet^+^, black). Data n = 6 mice per group, data compiled from two experiments, representative of three experiments. (**D**) Representative flow plot of ABCs (CD19^+^CD11c^+^T-bet^+^) in the spleen gated for YFP (γHV68.H2bYFP) and IFNγ with mean ± SEM. Dot plot displays the proportion of either uninfected (YFP^−^) or infected (YFP^+^) ABCs in the spleen that are positive for IFNγ. (**B,D**) Data presented as mean ± SEM, analyzed by Mann–Whitney test. Each data point represents an individual mouse. p-values indicated as asterisks as follows: ****p < 0.0001.

γHV68 is known to infect B cells, in particular germinal centre and memory B cells^32–35^. To determine if ABCs are directly infected by γHV68, we used a previously developed fluorescent strain of γHV68, γHV68.H2bYFP^36^ where fluorescence is easily detectable during acute infection, but over time falls off during latent infection. Mice were infected with γHV68.H2bYFP and 8 days p.i. spleens were collected, and flow cytometry was performed. We found that, during acute infection, a small proportion of ABCs were infected with γHV68. The same proportion of ABCs were positive for the fluorescent virus (1.2 ± 0.3%) as observed in previously activated B cells (1.1 ± 0.2%, Fig. 3A,B), demonstrating that ABCs are not preferably targeted for infection. To our knowledge this is the first evidence of a virus directly infecting ABCs. We next asked what proportion of the γHV68 reservoir is made up of ABCs. The primary reservoir for γHV68 is previously activated B cells which aligns with our results that 73% of γHV68-infected cells are IgD^−^ B cells (Fig. 3C). We found that ABCs make up only 6% of γHV68-infected cells (Fig. 3C). Other infected cell populations included innate cells such as macrophages and DCs, in alignment with previous findings^32^, as well as a small proportion of NK and T cells (Fig. 3C). We also examined the relationship between infection of ABCs and expression of IFNγ. We observed that during acute infection the proportion of ABCs expressing IFNγ was significantly lower in ABCs infected with γHV68 (4.3 $\pm$ 1.3%) as compared to uninfected ABCs (32.2 $\pm$ 1.0%, Fig. 3D). These results indicate that direct infection of ABCs was not driving IFNγ production and that at least during acute infection, ABCs are susceptible to virus-driven downregulation of IFNγ. These findings demonstrate that although a portion of ABCs are infected with γHV68, ABCs are not the major target for γHV68.

### ABCs are dispensable for the control of acute infection and establishment of latency

**Figure 4:**
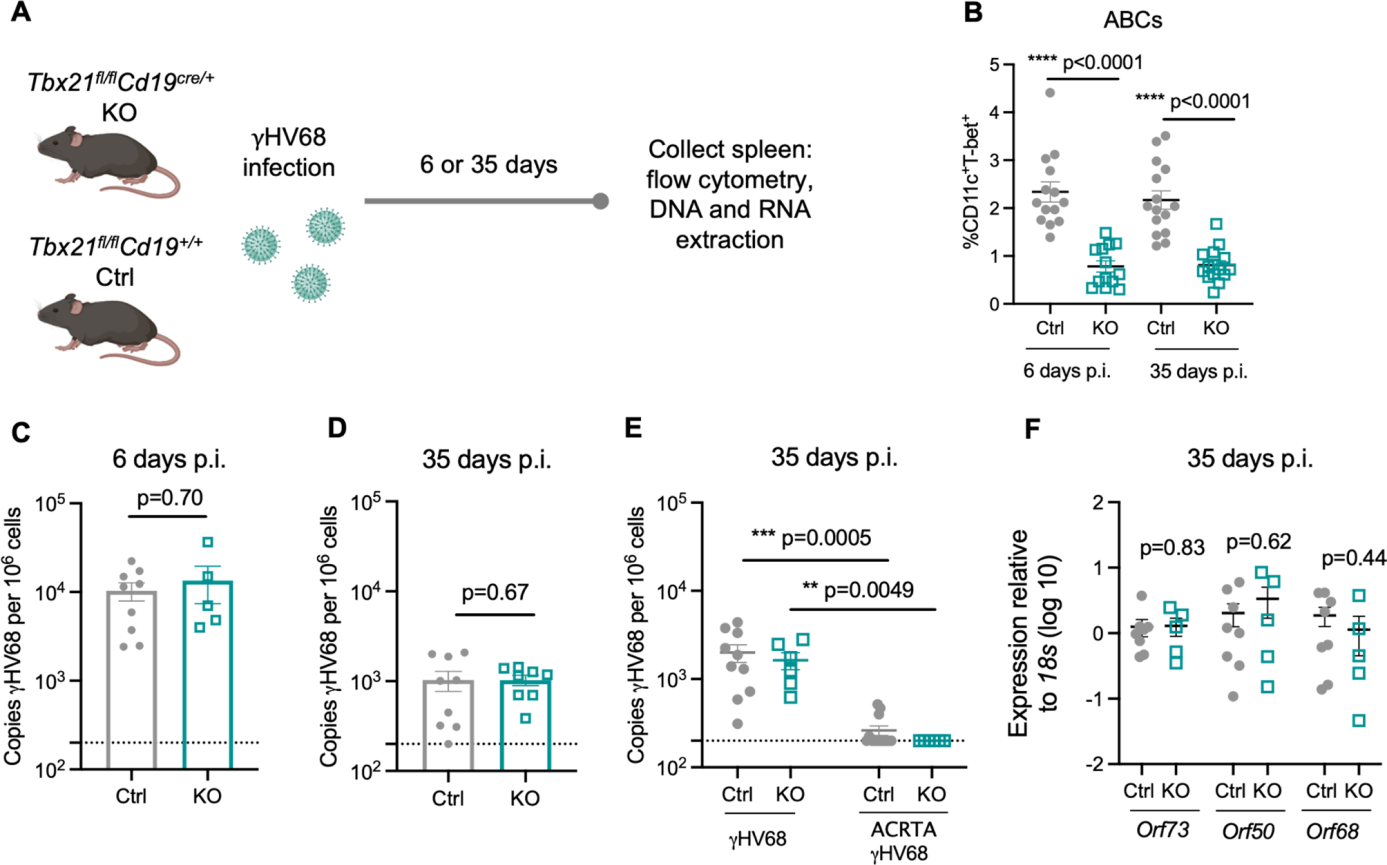
ABCs are not required for controlling lytic infection and achieving long-term latency. *Tbx21*^*fl/fl*^*Cd19*^+*/*+^ (Ctrl, filled grey circles) and *Tbx21*^*fl/fl*^*Cd19*^*cre/*+^ (KO, open blue squares) mice were infected i.p. with γHV68 or ACRTA-γHV68. 6 or 35 days p.i., spleens collected and processed for RNA extraction, DNA extraction, and flow cytometry. (**A**) Experimental scheme for data shown in panels (**B–D,F**). (**B**) Proportion of ABCs (CD11c^+^T-bet^+^) of previously activated B cells (CD19^+^IgD^−^) in the spleen in Ctrl and KO mice 6 and 35 days post-γHV68 infection. (**C,D**) Splenic γHV68 viral load (copies *Orf50* per million cells) in Ctrl (filled circles) and KO (open squares) mice as determined by qPCR day 6 and 35 p.i., with limit of detection indicated by dotted line. (**E**) Splenic γHV68 viral load (copies *Orf50* per million cells) in Ctrl and KO mice infected with γHV68 or ACRTA-γHV68 35 days p.i. (**F**) Relative expression in the spleen of *Orf73*, *Orf50*, and *Orf68* in Ctrl and KO mice at day 35 p.i. Each data point represents an individual mouse. Both male and female mice were included in the experiments. Data presented as mean ± SEM, analyzed by Mann–Whitney test (**B–D,F**) or Kruskal–Wallis H test (**E**).

To further examine the role(s) of ABCs in γHV68 infection, mice with a floxed B cell specific T-bet deletion were followed post-infection. Specifically, *Tbx21*^*fl/fl*^*Cd19*^*cre/*+^ (KO) and littermate *Tbx21*^*fl/fl*^*Cd19*^+*/*+^ (Ctrl) mice were infected with γHV68 for 6 or 35 days and the spleen was collected to examine the quantity of γHV68 by qPCR and the immune cell composition (Fig. 4A). Mice were genotyped by PCR and loss of ABCs in KO mice was confirmed by flow cytometry (Fig. 4B). No differences in clinical symptoms or weight changes were observed during γHV68 infection between Ctrl or KO mice (Fig. S4A).

To determine if knocking out ABCs alters immune cell composition, Ctrl and KO mice were infected or mock-infected with γHV68 and spleens analyzed at 6 or 35 days by flow cytometry to examine various T cell, B cell, and innate immune cell populations. No difference in the total number of splenocytes was observed (Fig. S4B). The composition of the splenic immune profile was similar between Ctrl and KO mice with no significant differences observed in the relative proportions of B cells, CD8 T cells, DCs, neutrophils, NK cells, or macrophages between mock-infected mice or those infected with γHV68 for 6 or 35 days, though KO mice had greater proportions of CD4 T cells than Ctrl mice 35 days post-infection (Fig. S5).

We then measured the quantity of γHV68 in the spleens of Ctrl and KO mice infected for 6 and 35 days by qPCR. We found no difference in that the quantity of γHV68 at 6 and 35 days p.i. between Ctrl and KO mice (Fig. 4C,D) and the quantity of γHV68 did not differ between male and female mice (Fig. S4C,D). To confirm that KO mice effectively control the lytic infection and develop latency, we infected Ctrl and KO mice with latency-deficient ACRTA-γHV68 for 35 days. We found that KO mice, like Ctrl mice, displayed similar viral loads that were near or below the limit of detection when infected with ACRTA-γHV68 (Fig. 4E). As no virus was detected at day 35, mice lacking ABCs were effectively controlling the lytic infection. Furthermore, we examined splenocytes from Ctrl and KO mice for the expression of genes associated with lytic infection (*Orf50*, *Orf68*) or latent infection (*Orf73*), and observed no differences in the relative expression of *Orf50, Orf68, or Orf73* between Ctrl and KO mice (Fig. 4F). *Orf50* encodes the replication and transcription activator protein, which initiates viral lytic gene expression^37^. *Orf68* encodes a packaging protein that assists in moving the newly replicated viral genomes to the packaging motor, where they are loaded into capsids^38^. *Orf73* encodes for the latency-associated nuclear antigen that is required for the establishment and maintenance of latent infection^39,40^. As such, equal expression of lytic and latency-associated genes *Orf50*, *Orf68,* and *Orf73* between Ctrl and KO mice suggests the harboring of similar levels of lytic and latent γHV68. Together, these results demonstrate that ABCs are not required for the clearance of acute infection and establishment of latency in steady-state conditions.

### Lack of ABCs results in a dysregulated γHV68 antibody response but does not alter the viral reservoir or anti-viral T cell response

**Figure 5:**
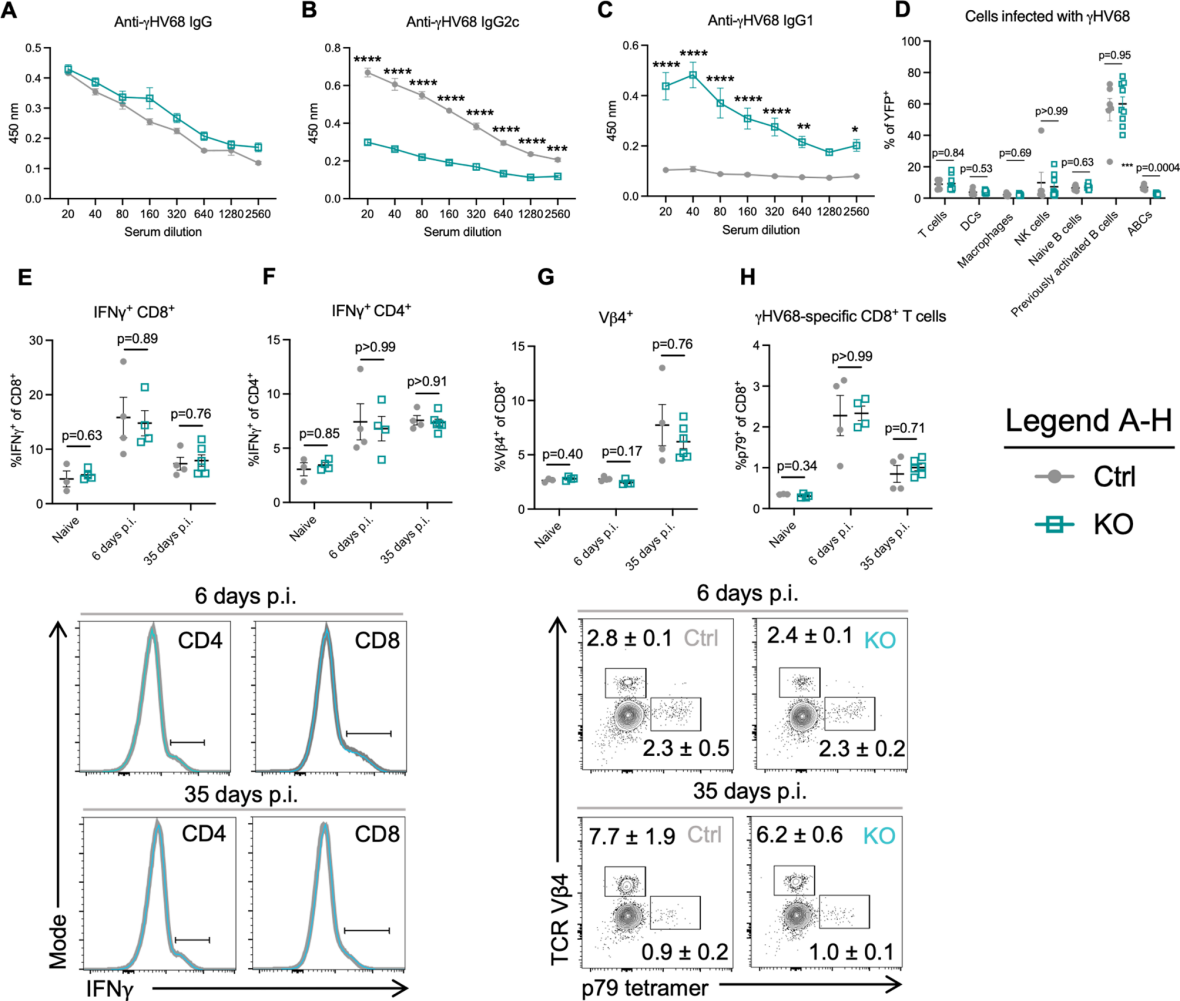
Mice without ABCs display a dysregulated anti-γHV68 antibody response, though no alterations to the cell populations infected with γHV68 or γHV68-responding T cells, compared to mice with ABCs. (**A–C**) *Tbx21*^*fl/fl*^*Cd19*^+*/*+^ (Ctrl, filled grey circles) and *Tbx21*^*fl/fl*^*Cd19*^*cre/*+^ (KO, open blue squares) mice were infected i.p. with γHV68 for 35 days. N = 5 per group, representative of 2 experiments. (**D**) Ctrl and KO mice were infected i.p. with γHV68.H2bYFP for 8 days, and various immune cell populations in the spleen analyzed for YFP expression. N = 6–8 per group, data compiled from 2 experiments. (**E–H**) Ctrl and KO mice were infected i.p. with γHV68 for 6 or 35 days, at which point spleens were collected for flow cytometry. Representative of 2 experiments. Flow cytometry plots are representative samples previously gated on live (**E,F**) CD45^+^CD3^+^ or (**G,H**) CD45^+^CD3^+^CD8^+^ splenocytes, with mean ± SEM. Each data point represents an individual mouse. Both male and female mice included in experiments. Data presented as mean ± SEM. Analyzed by two-way ANOVA (**A–C**) or Mann–Whitney test (**D–H**). p-values indicated as asterisks as follows: ****p < 0.0001, ***p < 0.001, **p < 0.01, *p < 0.05.

ABCs are known to secrete anti-viral IgG2a/c^7^, and transfer of serum into mice without ABCs during LCMV infection was partially able to restore control of the infection^16^. To examine the antibody response in Ctrl versus KO mice, mice were infected with γHV68 for 35 days and sera was collected. The levels of anti-γHV68 IgG were the identical between Ctrl and KO mice, though Th1-associated IgG2c was significantly decreased in KO mice, while Th2-associated IgG1 was significantly elevated, when compared to Ctrl mice (Fig. 5A–C). These results suggest that ABCs are the primary secretors of anti-γHV68 IgG2, though in their absence there is compensation by other B cell subsets resulting in increased anti-γHV68 IgG1 antibodies.

We next asked if the γHV68 reservoir was altered in mice without ABCs, as a portion of γHV68-infected cells were ABCs. In particular, we posited that an altered viral reservoir could impact the ability of KO mice to control γHV68 latency; previous findings indicate that various cell types infected by γHV68 may display differential susceptibility to reactivation^24,41^. To compare the viral reservoir in Ctrl and KO mice, we infected mice with a fluorescent strain, γHV68.H2bYFP, and, at 8 days p.i., examined immune cell populations that comprise the γHV68-infected population. We observed no difference in the proportion of infected cell populations, including T cells, DCs, macrophages, NK cells, and naïve and previously activated B cells (Fig. 5D). This suggests that the cell populations infected with γHV68 are not substantially altered in KO mice compared to Ctrl mice, making it unlikely that changes to the reservoir impacted susceptibility to viral reactivation.

To ask whether ABCs influence anti-viral immune cells in the spleen, we next examined three cell populations previously shown to be important for control of latent γHV68: IFNγ-producing cells, γHV68-specific CD8^+^ T cells, and Vβ4^+^CD8^+^ T cells. IFNγ is present at low levels during latency^42^ and is critical for controlling γHV68 reactivation from latency^24,25,43^. In particular, IFNγ-producing T cells are known to block γHV68 reactivation^24,44–46^. CD8^+^ T cells that harbor the Vβ4 TCR were also examined. CD8^+^Vβ4^+^ T cells expand following γHV68 infection and reach their highest levels during latency, wherein they persist throughout infection without taking on an exhausted phenotype^47–49^. γHV68-specific CD8^+^ T cells were also measured with tetramers to p79, an immunodominant γHV68 epitope for which CD8-specific memory T cells remain throughout latent infection^50^. We observed that the proportion of CD4^+^ and CD8^+^ T cells that express IFNγ were unchanged between mice with and without ABCs (Fig. 5E,F). Further, no differences were observed in the proportion of either Vβ4^+^CD8^+^ T cells or γHV68 p79-specific CD8^+^ T cells between mice with and without ABC circulating populations (Fig. 5G,H). Additionally, we found that the proportion of NK cells and B cells that express IFNγ, and the MFI of IFNγ on these populations, was not changed between Ctrl and KO mice whether mock-infected or infected for 6 or 35 days (Fig. S6). That a similar proportion of B cells expressed IFNγ indicates that another B cell subset likely compensates for the loss of IFNγ-expressing ABCs. Collectively, these findings indicate that ABCs are not acting to stimulate anti-viral immune cell populations in the spleen.

Together, these data indicate that knocking out ABCs leads to dysregulation of the γHV68 antibody response, without altering the γHV68 reservoir or T cell populations responding to the latent virus.

### In the absence of ABCs, reactivation of γHV68 occurs more readily following infections

**Figure 6:**
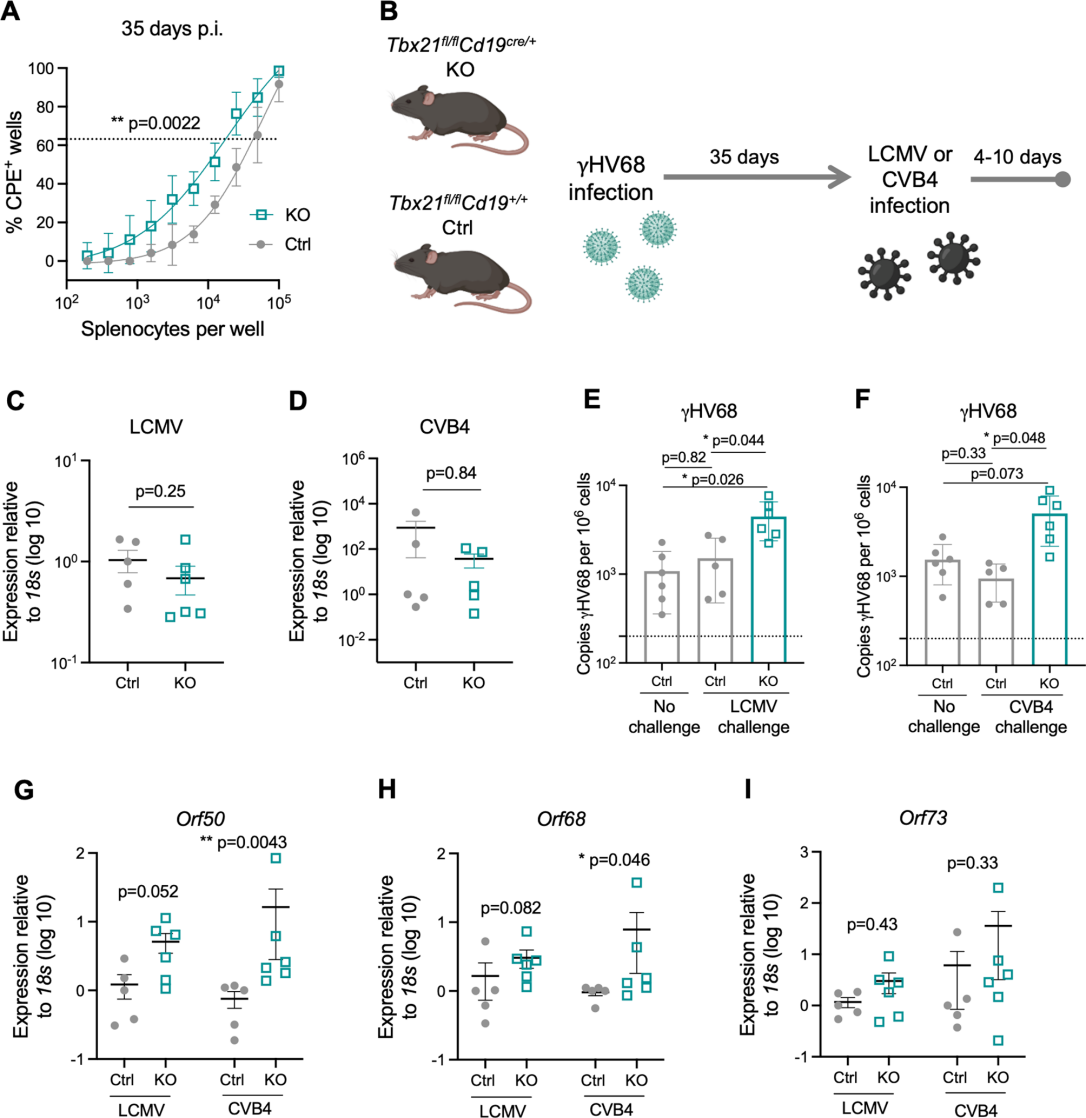
ABC knockout mice are more susceptible to reactivation. (**A**) *Tbx21*^*fl/fl*^*Cd19*^+*/*+^ (Ctrl, filled grey circles) and *Tbx21*^*fl/fl*^*Cd19*^*cre/*+^ (KO, open blue squares) mice were infected i.p. with γHV68. 35 days p.i., spleens were collected and processed for reactivation assay. N = 6 mice per group, data compiled from two independent experiments. (**B**) Experimental scheme for data shown in panels (**C–I**). Ctrl and KO mice were infected i.p. with γHV68 for 35 days and then challenged with either LCMV (Armstrong) or CVB4 for 10 to 4 days, respectively. (**C–I**) Spleens collected, processed, and DNA and RNA was extracted from total splenocytes following RBC lysis. (**C,D**) Relative expression of LCMV (**C**) and CVB4 (**D**) between Ctrl and KO mice measured by RT-qPCR. (**E,F**) Copies of γHV68 detected by qPCR in the spleen per million cells following challenge with LCMV or CVB4. (**G–I**) Relative expression in the spleen of *Orf73*, *Orf50*, and *Orf68* in Ctrl and KO mice. Each data point represents an individual mouse. Both male and female mice included in experiments. Data presented as mean ± SEM, analyzed by Mann–Whitney test (**A,C,D,G–I**) or one-way ANOVA (**E,F**). p-values indicated as asterixis as follows: **p < 0.01, *p < 0.05.

We posited that the persistence of ABCs long-term during γHV68 and their secretion of IFNγ, TNF, and anti-viral antibodies suggest that ABCs have an important role in the suppression of latent virus reactivation. To examine this role, ex vivo reactivation assays were performed on splenocytes from Ctrl and KO mice at 35 days p.i. Splenocytes from γHV68-infected KO mice demonstrated more frequent virus reactivation in culture compared to splenocytes from γHV68-infected Ctrl mice (Fig. 6A). This finding indicates that there is an increased propensity for γHV68 reactivation in the absence of ABCs, although the assay does not disentangle whether the influence is cell-intrinsic or extrinsic nor necessarily indicate in vivo reactivation. As we did not observe elevated viral load or expression of lytic-associated genes at day 35 p.i. in KO mice (Fig. 4D,F), we speculated that although mice lacking ABCs have elevated susceptibility to reactivation, they might be able to maintain latency in steady-state conditions, though additional stressors could result in reactivation in vivo.

Therefore, to further interrogate the role of ABCs in suppressing γHV68 reactivation, we next asked if mice without ABCs are more susceptible to γHV68 reactivation following infectious challenge. Importantly, we chose to examine the response to viruses that do not typically reactivate γHV68^51^, such as LCMV and coxsackievirus B4 (CVB4). These heterologous viral infections were performed as physiological mimics to ask if they were sufficient to cause reactivation of latent γHV68 in the absence of ABCs. Specifically, KO and Ctrl mice were infected with γHV68 for 35 days and then challenged with LCMV or CVB4 (Fig. 6B). No clinical symptoms were observed during either of the challenges in Ctrl or KO mice and there was no difference in the relative quantity of LCMV and CVB4 in the spleens of Ctrl versus KO mice (Fig. 6C,D). Following challenge with LCMV or CVB4, mice lacking ABCs had elevated quantities of γHV68 in the spleen compared to mice with ABCs (Fig. 6E,F). We reasoned that the increased γHV68 load following challenge may be due to increased reactivation and, in support, observed increased relative expression of two lytic-associated γHV68 genes, *Orf50* and *Orf68*, in KO mice compared to Ctrl mice following challenge with the heterologous viruses (Fig. 6G,H). Alternatively, we did not observe a difference in expression of the latency-associated gene *Orf73*, between Ctrl and KO mice (Fig. 6I). This also indicates that it is not simply an expansion in the number of latently infected B cells in the spleen and is a change in the active lytic expression of more virus. Further, we did not observe significant cell number increases in B cells between the KO and Ctrl mice post challenge (Fig. S7). These findings make clear that ABCs act to impede latent γHV68 reactivation in the face of mild stresses such as heterologous infection.

## Discussion

Here we have shown that ABCs are increased during acute γHV68 infection and persist during latency for at least 150 days. Previous studies have shown that ABCs persist following clearance of viral infections^10^ and during chronic infection^16^, though their contribution during a latent infection was unexplored. Our results demonstrate a novel role for ABCs: they continuously respond in an effector manner to latent viral infection by secreting anti-viral cytokines and antibodies. Further, our results indicate that ABCs may play an important role in suppressing γHV68 reactivation during subsequent heterologous infectious challenges.

Our results show that ABCs continuously expresses anti-viral cytokines during latency and the presence of the latent virus is required for the sustained anti-viral cytokine expression by ABCs. Compared to other B cells, ABCs display uniquely high expression of anti-viral cytokines IFNγ and TNF during γHV68. In addition to expressing cytokines, ABCs also produce anti-viral antibodies, as deficiency of ABCs results in a significant loss of γHV68-specific IgG2c antibodies. We hypothesized that the production of anti-viral cytokines and antibodies by ABCs could be a way in which ABCs restrain γHV68 reactivation. Using mice deficient in ABCs, we showed that ABCs are important for the suppression of γHV68 reactivation in the face of heterologous viral challenge.

A surprising finding of our study is that ABCs are increased more so in female than male mice throughout γHV68 infection, in both the blood and spleen. Sex-differences are well-documented in anti-viral immune responses^52^ and ABCs have previously been shown to display a female sex bias in contexts of aging and autoimmunity^4,53^. It has recently been shown that the ABC female sex bias in lupus mice is abolished following the duplication of *Tlr7* in male mice^53^. While we show that there is no difference in the quantity of γHV68 between female and male mice, TLR7 stimulation is known to be important for ABC differentiation^3,4^ and TLR7 is critical for the control of lytic γHV68 infection and maintenance of latency^54^. While increased frequencies of ABCs were observed in female mice compared to males, we found that a similar proportion of the ABCs express anti-viral cytokines in both males and females. No differences between male and female mice were observed in viral load or reactivation.

Using a parallel approach to knockout T-bet in B cells with bone marrow chimeras, it was previously shown that mice lacking ABCs display an increased quantity of γHV68 during the early stage of latency, at day 18 p.i., compared to controls^7^. This difference with our results is most easily explained by the fact that the two studies measure viral titre at different timepoints (day 18 p.i., the early-stage latency versus day 35 p.i., long-term latency). These two timepoints are distinct, with viral reactivation occurring more frequently at early-stage compared to long-term latency^28^. Additionally, it is well known that γHV68 latency establishment can be delayed by certain immunological perturbations, such as loss of transcription factor NF-κB^55^. Therefore, we propose the possibility that mice without ABCs display a delayed establishment of latency and/or elevated viral reactivation during early latency under steady-state conditions, resulting in elevated viral titers at an early latency timepoint, though no difference during long-term latency. Our results also highlight elevated viral reactivation ex vivo during long-term latency and demonstrate a loss of latency maintenance in the face of heterologous challenge. The results of these two studies together indicate that mice lacking ABCs display elevated viral reactivation and hint at potential differences in the kinetics of latency establishment in the absence of ABCs. Additionally, these two studies differ in the inoculating dose (lower herein) and the method of ABC knockout, which could also contribute to the observed differences.

ABCs are known to possess an array of functional capacities and the precise mechanism(s) of ABC contribution to the restraint of γHV68 reactivation will be the focus of future studies. Here we have shown that only the antibody response is altered in mice deficient in ABCs and unexpectedly, changes are not observed in the anti-viral T cell response or viral reservoir. The ability of ABCs to continuously secrete anti-viral cytokines during latent infection is an additional mechanism that likely contributes to the suppression of γHV68 reactivation. Understanding the precise localization of ABCs in relation to γHV68-infected B cells will also aid in our understanding of the development of splenic structures, in particular germinal centres, as ABCs have been shown to play a role in their development^56^. In future it will also be worthwhile to perform intranasal inoculations, in comparison to the intraperitoneal route that was used here, to investigate a potential role of ABCs at mucosal barriers.

Whether ABCs play a role in suppressing the reactivation of human gamma-herpesvirus infections such as Epstein-Barr virus (EBV) and Kaposi sarcoma-associated herpesvirus, is not known. Understanding this role would have important relevance in diseases associated with these human gammaherpesviruses, including various malignancies, myalgic encephalomyelitis/chronic fatigue syndrome, and chronic-active EBV^57–59^.

This work demonstrates that ABCs are a long-lasting effector population during γHV68 latent infection. ABCs are likely one of many players in the maintenance of latent infection and have a unique role in the control of latent virus reactivation following subsequent heterologous infections.

## Methods

### Mice

*Tbx21*^*fl/fl*^*Cd19*^*cre/*+^ mice were generated by crossing *Tbx21*^*fl/fl*^*Cd19*^*cre/*+^ and *Tbx21*^*fl/fl*^*Cd19*^+*/*+^ mice with *Tbx21*^*fl/fl*^ and *Cd19*^*cre/*+^ mice provided by Dr. Pippa Marrack^56^. C57BL/6(J) mice were originally purchased from The Jackson Laboratory and all animals were bred and maintained under specific-pathogen free conditions in the animal facility at the University of British Columbia. Mice were housed in individually ventilated cages in groups of up to five animals with unrestricted access to food and water. All animal work was approved by the Canadian Council for Animal Care (Protocols A17- 0105, A17-0184) and performed in accordance with relevant guidelines and regulations, following recommendations in the ARRIVE guidelines.

### γHV68, ACRTA-γHV68, and γHV68H2B.YFP infection

γHV68 WUMS strain (ATCC), ACRTA-γHV68 (developed by Dr. Ting-Ting Wu, gift of Dr. Marcia A. Blackman)^31^, and γHV68H2B.YFP (developed and provided by Dr. Samuel H. Speck) were propagated in Baby Hamster Kidney cells (BHK, ATCC). Viruses were diluted in Minimum Essential Media (MEM) prior to infection and maintained on ice. 6- to 8-week-old mice were infected i.p. with 10^4^ PFU of γHV68, ACRTA-γHV68, γHV68H2B.YFP, or mock-infected with MEM as previously described. Clinical symptoms were not observed during γHV68, ACRTA-γHV68, or γHV68H2B.YFP infections in C57BL/6(J) nor *Tbx21*^*fl/fl*^*Cd19*^*cre/*+^ or *Tbx21*^*fl/fl*^*Cd19*^+*/*+^ mice.

### LCMV infection

LCMV Armstrong strain 53b (originally acquired from Dr. M.B. Oldstone) was propagated on BHK cells. Prior to infection, virus was diluted in RPMI-1640 media (Gibco) and maintained on ice. Mice (11- to 13-week-old) were infected i.p. with 2 × 10^5^ PFU LCMV or mock infected with media. No clinical symptoms were observed from LCMV infection in *Tbx21*^*fl/fl*^*Cd19*^*cre/*+^ nor *Tbx21*^*fl/fl*^*Cd19*^+*/*+^ mice.

### CVB4 infection

CVB4 Edward strain was propagated on HeLa cells and titred by plaque assay. Virus was diluted in Dulbecco’s Modified Eagle Medium (DMEM, Gibco) and maintained on ice prior to infection. Mice (11- to 13-week-old) were infected i.p. with 100 PFU CBV4. No clinical symptoms were observed from CVB4 infection in *Tbx21*^*fl/fl*^*Cd19*^*cre/*+^ or *Tbx21*^*fl/fl*^*Cd19*^+*/*+^ mice.

### Tissue harvesting and processing for flow cytometry

Mice were anesthetised with isoflurane and euthanized by cardiac puncture. For flow cytometry, blood was collected by cardiac puncture into 100 μl 0.5 M Ethylenediaminetetraacetic acid (EDTA) to prevent clotting and placed on ice until processing. Spleen extracted and placed into 2 ml PBS and kept on ice until processing. Blood was incubated in 10 ml warmed (37 °C) ACK lysis buffer for 15 min at room temperature to remove red blood cells and washed twice with FACS buffer. Spleens were mashed through a 70 μm cell strainer with a 3 ml syringe insert to make a single cell suspension for each sample. Splenocytes were incubated in 4 ml warmed (37 °C) ACK lysis buffer for 10 min on ice to lyse red blood cells and remaining cells were resuspended in FACS buffer and kept on ice until further use.

### Flow cytometry analysis of cell-type specific surface antigens and intracellular cytokines

For analysis of extracellular and intracellular antigens, 2 million cells per spleen sample or all cells collected per blood sample were stained with appropriate antibodies. Prior to staining, samples were incubated at 4 °C covered from light for 30 min with 2 μl/ml Fixable Viability Dye eFluor506 (Thermo Fisher) while in PBS and then resuspended in rat anti-mouse CD16/32 (Fc block, BD Biosciences) antibody for 10 min. Then, samples were stained with fluorochrome labeled antibodies (Table 1) against cell surface antigens for 30 min covered from light at 4 °C, washed, and resuspended in Fix/Perm buffer (Thermo Fisher) for 30 min-12 h while maintained covered from light at 4 °C. Samples were then washed twice with perm buffer and incubated 40 min with antibodies for intracellular antigens (Table 1) in Perm buffer at covered from light at RT. Cells were then washed and resuspended in FACS buffer with 2 mM EDTA. For analysis of cytokine production, 4 million cells per sample were stimulated ex vivo for 3 h at 5% CO2 at 37 °C in Minimum Essential Media (Gibco) containing 10% fetal bovine serum (FBS, Sigma-Aldrich), 1 μl/ml GolgiPlug (BD Biosciences), 10 ng/ml PMA (Sigma-Aldrich) and 500 ng/ml ionomycin (Thermo Fisher) and washed prior to staining. Samples were collected on an Attune NxT Flow Cytometer (Thermo Fisher) and analyzed with FlowJo software v10 (FlowJo LLC).

**Table 1:** Flow cytometry antibodies.

Antigen	Fluor	Clone	Dilution	Company
CD3e	eFluor 450	500A2	1:200	Thermo Fisher Scientific
CD19	PE	1D3	1:200	Thermo Fisher Scientific
CD19	Pe-Cy7	1D3	1:200	Thermo Fisher Scientific
CD19	SB780	1D3	1:100	Thermo Fisher Scientific
IgD	Pe-Cy7	11–26	1:200	Thermo Fisher Scientific
CD11c	Alexafluor700	N418	1:100	Thermo Fisher Scientific
CD11c	APC	N418	1:100	Biolegend
T-bet	PerCpCy5.5	4B10	1:100	Thermo Fisher Scientific
IFNγ	APC	XMG1.2	1:100	Thermo Fisher Scientific
TNF	Pe-Cy7	MP6-XT22	1:100	Biolegend
IL-17A	PEDazzle594	TC11-18H10.1	1:100	Biolegend
CD45	Alexafluor700	30-F11	1:200	Thermo Fisher Scientific
CD45	APC-eFluor 780	30-F11	1:200	Thermo Fisher Scientific
CD4	SB645	RM4-5	1:100	Thermo Fisher Scientific
CD8	APC-eFluor 780	53–6.7	1:200	Thermo Fisher Scientific
CD8	PEDazzle594	53–6.7	1:100	Biolegend
CD11b	Pe-Cy7	M1/70	1:100	Thermo Fisher Scientific
Ly6G	PerCpCy5.5	RB6-8C5	1:100	Thermo Fisher Scientific
CD335	FitC	29A1.4	1:100	Thermo Fisher Scientific
NK1.1	PE	PK136	1:100	Thermo Fisher Scientific
F4/80	APC-eFluor 780	BM8	1:100	Thermo Fisher Scientific
CD62L	PerCpCy5.5	MEL-14	1:100	Thermo Fisher Scientific
CD44	Alexafluor700	IM7	1:200	Thermo Fisher Scientific
PD1	PerCpeFluor 710	RMP1-30	1:100	Thermo Fisher Scientific
CXCR5	PE	SPRCL5	1:100	Thermo Fisher Scientific
FoxP3	Alexafluor700	FJK-16s	1:100	Thermo Fisher Scientific
CD138	PEDazzle594	281-2	1:100	Biolegend
CD95	FitC	SA367H8	1:100	Biolegend
IgM	APCCy7	RMM-1	1:100	Biolegend
TCR Vβ4	FitC	KT4	1:100	BD Bioscience

### Tetramer staining

γHV68-specific CD8 T cells were identified by staining with a tetramer acquired from the NIH Tetramer Facility. 2 million cells per well were incubated for 1 h at RT with p79 (K^b^/ORF61_524–531_ TSINFVKI, diluted 1:400) following viability staining and Fc receptor block, as described above, but before extracellular antigen staining. Uninfected mice stained with tetramers used as negative gating controls.

### γ*HV68 qPCR*

DNA was isolated from 4 × 10^6^ splenocytes using PureLink™ Genomic DNA Mini Kit (Thermo Fisher) and quantified with a spectrophotometer. qPCR was performed using 2 × QuantiNova Probe Mastermix (Qiagen, USA) on the Bio-Rad CFX96 Touch™ Real Time PCR Detection system. Copies of γHV68 were quantified in duplicate wells with 150 ng DNA per reaction using primers and probes specific to γHV68 *Orf50* and mouse *Ptger2* (Table 2). Standard curves were obtained by serial dilutions of *Orf50* and *Ptger2* gBlocks (*Orf50*: 2 × 10^6^–2 × 10^1^; *Ptger2*: 5 × 10^7^–5 × 10^2^) that were amplified in parallel.

**Table 2:** γHV68 qPCR primers and probe sequences.

Gene	Sequence
*Ptger2* Forward Primer	5′-TACCTTCAGCTGTACGCCAC-3′
*Ptger2* Reverse Primer	5′-GCCAGGAGAATGAGGTGGTC-3′
*Ptger2* Probe	5′-/56-FAM/CCTGCTGCT/ZEN/TATCGTGGCTG/3IABkFQ/-3′
*Orf50* Forward Primer	5′-TGGACTTTGACAGCCCAGTA-3′
*Orf50* Reverse Primer	5′- TCCCTTGAGGCAAATGATTC-3′
*Orf50* Probe	5′-/56-FAM/TGACAGTGC/ZEN/CTATGGCCAAGTCTTG/3 IABkFQ/-3′

### RT-qPCR for γHV68 lytic and latency-associated genes and relative quantity of LCMV and CVB4

Portion of spleen stabilized in RNAlater immediately following collection and stored at − 80 °C for up to 12 weeks. RNA extracted with RNeasy mini kit (Qiagen, cat no. 74104) and cDNA immediately synthesized with High-Capacity cDNA Reverse Transcription Kit (Thermo Fisher, cat no. 4368814). Reaction performed with 500 ng per reaction in duplicate wells and cDNA quantified with a spectrophotometer. qPCR was performed using iQTM SYBR® Green supermix (Bio-Rad) on the Bio-Rad CFX96 Touch™ Real Time PCR Detection system. Transcript specific primers have been previously described for *Orf50*, *Orf73*, and *Orf68*^60^, LCMV^61^, and CVB4^62^ and ordered from Integrated DNA Technologies (Table 3). *Orf50, Orf73, and Orf68* expression normalized to the ribosomal housekeeping gene *18 s* using the 2^–∆∆Ct^ method with expression determined relative to median value in Ctrl group.

**Table 3:** qPCR for lytic and latent γHV68 genes primer sequences.

Gene	Sequence
*Orf50* forward	5′-GGCCGCAGACATTTAATGAC-3′
*Orf50* reverse	5′-GCCTCAACTTCTCTGGATATGCC-3′
*Orf73* forward	5′-AAGGGTTGTCTTGGCCTAC TGTG-3′
*Orf73* reverse	5′-AGAGATGCTGTGGGACCATGTTG-3′
*Orf68* forward	5′-CTCAAATACACTGGCCGCCATC-3′
*Orf68* reverse	5′-CGTGCTTGAGATATGAGTGAGT-3′
*CVB4* forward	5′-CCCACAGGACGCTCTAATA-3′
*CVB4* reverse	5′-CAGAGTTACCCGTTACGACA-3′
*LCMV GP* forward	5′-TGCCTGACCAAATGGATGATT-3′
*LCMV GP* reverse	5′-CTGCTGTGTTCCCGAAACACT-3′
*18s* forward	5′-GTAACCCGTTGAACCCCATT-3′
*18s* reverse	5′-CCATCCAATCGGTAGTAGCG-3′

### Ex vivo limiting dilution reactivation assay

A single cell suspension following RBC lysis with ACK lysis buffer was prepared from freshly collected spleens. To analyze ex vivo reactivation, limiting dilution reactivation assay was performed as previously described^18^. Twofold serial dilutions of splenocytes starting at 10^5^ cells per well were plated onto monolayer of mouse embryonic fibroblast (MEF, C57BL/6) cells (ATCC) in 96-well flat-bottom polystyrene tissue culture plates. Twelve wells were plated per dilution. Plates were incubated at 37 °C 5% CO_2_ for 10 days and scored for microscopically for viral cytopathic effects (CPE). Data were plotted as sigmoidal dose curves and interpolation was used to determine the cell density per sample at which 63.2% of wells were positive for CPE.

### Anti-γHV68 antibodies

Serum was collected via cardiac puncture and maintained at RT to allow for clotting. The sera were isolated by centrifugation 2000×*g* for 10 min, aliquoted, and stored for up to 12 months at − 80 °C prior to running the ELISA. Anti-γHV68 antibodies were quantified by standard indirect ELISA. Briefly, γHV68 virions were inactivated in 4% paraformaldehyde (PFA) for 20 min at RT. Then, ELISA plates (NUNC, Thermo Fisher) were coated with 5 μg/ml γHV68 in coating buffer (0.05 M NaHCO_3_, pH 9.6) with 1% PFA overnight at 4 °C. The plate was then washed 4× with wash buffer (PBS-0.05% Tween-20), blocked in blocking buffer (5% NBCS in PBS) for 1 h at 37 °C, and incubated with serial dilutions (1:20, 1:40, 1:80, 1:160, 1:320, 1:640, 1:1280, 1:2560) of test sera diluted in blocking buffer for 2 h at 37 °C. The plate was washed 4× with wash buffer and bound antibody was incubated with HRP-conjugated goat anti-mouse IgG (Thermo Fisher), rat anti-mouse IgG1 (BD Biosciences), or goat anti-mouse IgG2c (Thermo Fisher), all diluted 1:500 in blocking buffer, for 1 h at 37 °C, washed 4× with wash buffer, and detected by TMB substrate (BD Biosciences). Absorbance was read at 450 nm on a VarioSkan Plate Reader (Thermo Fisher).

### Statistical analyses

Data analysis and presentation as well as statistical tests were performed using GraphPad Prism software 8.4.2 (GraphPad Software Inc.). Results are presented as mean ± SEM. Statistical tests, significance (p-value), sample size (n, number of mice per group) and number of experimental replicates are stated in the figure legends. Statistical analyses included: two-way ANOVA with Geisser-Greenhouse’s correction, Mann–Whitney test, and one-way ANOVA. P-values indicated by asterisks as follows: ****p < 0.0001, ***p < 0.001, **p < 0.01, *p < 0.05.

### Study approval

All work was approved by the Animal Care Committee (ACC) of the University of British Columbia (Protocols A17-0105, A17-0184).

## Supplementary Information

Supplementary Figures.

## Data Availability

All data generated or analysed during this study are included in this published article and its Supplementary Information files.
